# A multiplex one-tube nested real time RT-PCR assay for simultaneous detection of respiratory syncytial virus, human rhinovirus and human metapneumovirus

**DOI:** 10.1186/s12985-018-1061-0

**Published:** 2018-10-30

**Authors:** Zhi-shan Feng, Li Zhao, Ji Wang, Fang-zhou Qiu, Meng-chuan Zhao, Le Wang, Su-xia Duan, Rui-qing Zhang, Chen Chen, Ju-Ju Qi, Tao Fan, Gui-xia Li, Xue-jun Ma

**Affiliations:** 1 Children’s Hospital of Hebei Province, Shijiazhuang, 050031 Hebei China; 2 Hebei Medical University, Shijiazhuang, 050031 Hebei China; 3 Key Laboratory for Medical Virology, National Health and Family Planning Commission, National Institute for Viral Disease Control and Prevention, Chinese Center for Disease Control and Prevention, No. 155 Changbai Street, Chang ping District, Beijing, 102206 China

**Keywords:** RSV, HRV, HMPV, Detection, LNA, Multiplex one-tube nested real-time RT-PCR

## Abstract

**Background:**

Respiratory syncytial virus (RSV), human Rhinovirus (HRV) and human Metapneumo Virus (HMPV) are important viral pathogens causing acute respiratory tract infections in the hospitalized patients. Sensitive and accurate detection of RSV, HRV and HMPV is necessary for clinical diagnosis and treatment.

**Results:**

A locked nucleic acid (LNA)-based multiplex closed one-tube nested real-time RT-PCR (mOTNRT-PCR) assay was developed for simultaneous detection of RSV, HRV and HMPV. The sensitivity, specificity, reproducibility and clinical performance of mOTNRT-PCR were evaluated and compared with individual real time PCR (RT-qPCR) assay using clinical samples. The analytical sensitivity of mOTNRT-PCR assay was 5 copies/reaction for RSV, HRV and HMPV, respectively, and no cross-reaction with other common respiratory viruses was observed. The coefficients of variation (CV) of intra-assay and inter-assay were between 0.51 to 3.67%. Of 398 nasopharyngeal aspirates samples tested, 109 (27.39%), 150 (37.69%) and 44 (11.06%) were positive for RSV, HRV and HMPV, respectively, whereas 95 (23.87%), 137 (34.42%) and 38 (9.55%) were positive for RSV, HRV and HMPV, respectively, by individual RT-qPCR assay. Thirty three samples that were positive by mOTNRT-PCR but negative by RT-qPCR were confirmed as true positives by sequencing using reported traditional two-step nested PCR assay.

**Conclusion:**

mOTNRT-PCR assay reveals extremely higher sensitivity than that of RT-qPCR assay for detecting RSV, HRV and HMPV in clinical settings.

**Electronic supplementary material:**

The online version of this article (10.1186/s12985-018-1061-0) contains supplementary material, which is available to authorized users.

## Background

Acute respiratory tract infections causes high morbidity and mortality in hospitalized patients and nearly 80% of infections are viral [1–3]. Respiratory syncytial virus (RSV), human rhinovirus (HRV) and human metapneumovirus (HMPV) are the main causative agents of acute respiratory tract infections in humans worldwide across all age group [4–6]. RSV and HMPV are negative-sense single-stranded RNA viruses of the family Pneumoviridae. HRV is a small and positive single-stranded RNA virus belonging to the Picornaviridae family. RSV, HMPV and HRV can cause disease ranging from mild upper respiratory infection to more severe lower respiratory infection in children and adults. In particularly, the young children, the elderly, severely immunecompromised adults are at high risk of serious disease following RSV, HMPV or HRV infection [4–7]. Thus, early and accurate detection of RSV, HRV and HMPV is extremely important for clinical diagnosis and treatment.

Multiplex PCR or traditional real time PCR (RT-qPCR) assays have been developed worldwide for the detection of RSV, HRV and HMPV [8–14]. However, the limit of detection of these assays is not yet adequate for assessing clinical specimens with a low viral load. In the present study, we adopted the design of locked nucleic acid (LNA)-modified primers and develop a multiplex one-tube nested real time RT-PCR (mOTNRT-PCR) assay for simultaneous detection of RSV, HRV and HMPV with the advantages of higher sensitivity, more convenience and cost-effectiveness than individual RT-qPCR.

## Results

### Sensitivity and specificity for mOTNRT-PCR assay

**Fig. 1:**
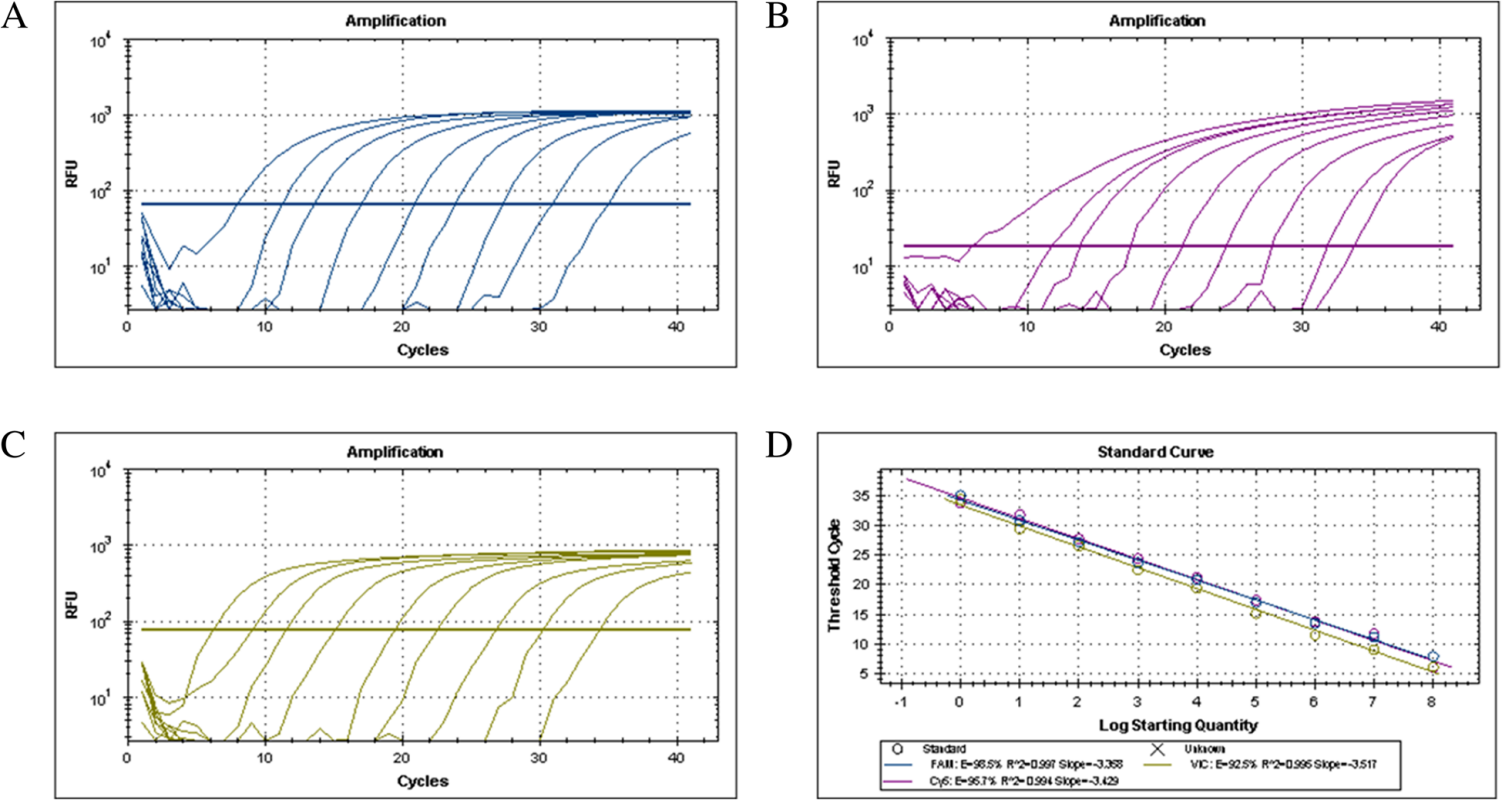
The sensitivity analysis and standard curves of the mOTNRT-PCR assay. **a**, **b**, and **c** are the amplification curves of RSV, HRV and HMPV, respectively using serial 10-fold dilutions of the mixed recombinant plasmids from 10^8^ to 10^0^ copies/μL, and **d** is the standard curves of the mOTNRT-PCR assay. The R^2^ of RSV, HRV and HMPV were 0.997, 0.994, 0.995 and the E of RSV, HRV and HMPV were 98.5, 95.7 and 92.5%, respectively

As shown in (Fig. 1a, b and c), the sensitivities of mOTNRT-PCR assay for RSV, HRV and HMPV were 5 copies /reaction. The standard curves acquired from the mOTNRT-PCR was shown in (Fig. 1d). The correlation coefficient (R^2^) of the standard curves of RSV, HRV and HMPV in multiplex reaction were 0.997, 0.994 and 0.995, respectively. The amplification efficiencies (E) for the different targets were 98.5, 95.7 and 92.5%, respectively, which was considered as acceptable for a multiplex screening assay.

A total of 159 RSV, HRV and HMPV-negative by mOTNRT-PCR out of 398 clinical specimens were used to determine the specificity of mOTNRT-PCR in this study. These samples were previously tested [15] and confirmed to be positive for either of a variety of other respiratory pathogens including, parainfluenza virus (PIV), mycoplasma pneumonia (MP), Chlamydia (including CP and CT), human Bocavirus (HBOV), adenovirus (ADV), coronavirus (COV), Chlamydia, influenza virus types A (Flu A), influenza virus FluA-H3, FluA-H1N1, and influenza virus types B (Flu B). No unspecific amplification or detection of mOTNRT-PCR was observed with these specimens (data not shown), suggesting the high specificity of mOTNRT-PCR assay.

### Reproducibility of the mOTNRT-PCR assay

Three different concentration mixtures of 10^6^, 10^4^ and 10^2^ copies/μL of each standard plasmid were respectively tested in triplicates within the same run. The inter-assay variability was evaluated by testing three dilutions of plasmids (10^6^,10^4^ and 10^2^ copies/μL) on three different days within 1 week. The CV values of intra-assay and inter-assay were 0.51–3.66% (Table 1) and 0.83–3.67% (Table 2), respectively. Overall, these results show that the assay is reliable with different Ct values.

**Table 1:** Intra-assay of the mOTNRT-PCR

	Target	Conc. (copies/reaction)	Number of determinations	Mean Ct	SD	CV (%)
Intra-assay	RSV	5 × 10^6^	3	13.23	0.48	3.66
		5 × 10^4^	3	19.90	0.13	0.64
		5 × 10^2^	3	26.77	0.42	1.57
	HRV	5 × 10^6^	3	13.89	0.10	0.74
		5 × 10^4^	3	21.60	0.29	1.36
		5 × 10^2^	3	27.88	0.14	0.51
	HMPV	5 × 10^6^	3	11.83	0.40	3.39
		5 × 10^4^	3	19.73	0.26	1.33
		5 × 10^2^	3	27.32	0.55	2.01

**Table 2:** Inter-assay of the mOTNRT-PCR

	Target	Conc. (copies/reaction)	Number of determinations	Mean Ct	SD	CV(%)
Inter-assay	RSV	5 × 10^6^	3	14.19	0.52	3.67
		5 × 10^4^	3	20.49	0.65	3.19
		5 × 10^2^	3	26.86	0.22	0.83
	HRV	5 × 10^6^	3	14.21	0.27	1.87
		5 × 10^4^	3	21.38	0.41	1.91
		5 × 10^2^	3	28.04	0.60	2.13
	HMPV	5 × 10^6^	3	12.34	0.43	3.45
		5 × 10^4^	3	19.29	0.37	1.91
		5 × 10^2^	3	27.75	0.33	1.20

### Clinical performance of the mOTNRT-PCR vs the RT-qPCR

Totally, 398 nasopharyngeal aspirates (NPAs) selected from children hospitalized with respiratory infection was examined by mOTNRT-PCR assay and previously published RT-qPCR assays [8–10]. mOTNRT-PCR assay detected 109 (27.39%) RSV infections with the Ct range from 12.37 to 35.00, 150 (37.69%) HRV with the Ct range from 12.70 to 33.49 and 44 (11.06%) HMPV with the Ct range from 10.79 to 35.00. Among them, 62 (15.58%) virus co-infections were found, including 36 (9.05%) RSV and HRV, 7 (1.76%) RSV and HMPV, 17 (4.27%) HRV and HMPV and 2 (0.50%) RSV, HRV and HMPV, respectively. Whereas RT-qPCR detected 95 (23.87%) RSV infections with the Ct range from 17.04 to 39.56, 137 (34.42%) HRV with the Ct range from 19.52 to 34.59 and 38 (9.55%) HMPV with the Ct range from 13.23 to 38.04. The comparisons between the results of two assays are summarized in Table 3, showing 96.48%, 96.73% and 98.49% agreements in detecting RSV, HRV and HMPV, respectively. Compared with the RT-qPCR assays, the specificities of the mOTNRT-PCR assay in detection of RSV, HRV and HMPV were more than 95% and the sensitivities of the mOTNRT-PCR assay were 100%. Thirty three samples that were positive by mOTNRT-PCR but negative by RT-qPCR were confirmed as true positives by sequencing using the traditional two-step nested PCR assay [16]. The detailed sequencing results were shown in Additional files 1, 2 and 3.

**Table 3:** Comparison of mOTNRT-PCR and RT-qPCR assays for detecting 398 clinical samples

Virus	No. of samples with result mOTNRT-PCR vs RT-qPCR				Performance of mOTNRT-PCR compared with RT-qPCR		
	+/+	+/−	−/+	−/−	Sensitivity(%)	Specificity(%)	Concordance(%)
RSV	95	14	0	289	100	95.38	96.48
HRV	137	13	0	248	100	95.02	96.73
HMPV	38	6	0	354	100	98.33	98.49

## Discussion

In recent years, many ordinary multiple PCR and mono RT-qPCR assays have been routinely applied in the well-equipped laboratory for the diagnosis of RSV, HRV and HMPV. Mono RT-qPCR [11, 12, 14] cannot simultaneously detect RSV, HRV and HMPV infections. Multiple PCR assays [8, 13] enable detection of co-infection but are either more likelihood of cross-contamination or insensitive. The two-step nested PCR assay has the high sensitivity [16], however, is more likelihood of cross-contamination. LNA modification can be performed on any base of the oligonucleotide strand resulting in increased melting temperature and improved amplification sensitivity and specificity [17]. LNA has been used in many applications including the detection of single nucleotide polymorphism (SNP) analyses [18], real-time PCR probes [19], microarray probes [20] and PCR primers [17]. In this study, we used LNA-modified primers to develop a mOTNRT-PCR assay for simultaneous detection of RSV, HRV and HMPV. To our best knowledge, this is the first report on the simultaneous detection of RSV, HRV and HMPV in a closed one-tube using LNA.

In the mOTNRT-PCR assay, the LNA-modified outer primers of RSV, HRV and HMPV had higher annealing temperatures than the inner primers thus possibly allowing an independent reaction during the amplification. The purpose of this design was to maximize the difference in the annealing temperatures (64 °C vs 54 °C) of the outer primer modified by LNA and inner primer sets, allowing one-step nested amplification to be carried out. The 10 °C difference in the melting temperature between outer and inner primers was applicable for developing a one-tube nested PCR successfully in our previous reports [21, 22]. According to the rule of TSP [21, 22], the LNA-outer primers were carried out at a high annealing temperature (64 °C) in the initial cycles of the mOTNRT-PCR, enabling hybridization of only the outer primers, and later cycles were carried out at a lower annealing temperature (54 °C), which enables hybridization of both the inner primers to the amplicons and the antisense oligonucleotides to the outer primers.To minimize the primer competition and ensure that the final amplification products were inner primer products of RSV, HRV and HMPV, the working concentration of each primer were optimized.The reaction conditions of the mOTNRT-PCR assay were also optimized, and this enabled the detection of RSV, HRV and HMPV with extremely high sensitivity (5copies /reaction) while maintaining good specificity as shown in this study (Fig. 1).

This mOTNRT-PCR assay was further evaluated and compared with RT-qPCR assay using 398 clinical samples. As shown in Table 3, the positive sample numbers of RSV, HRV and HMPV detected by RT-qPCR were 95 (23.87%), 137 (34.42%) and 38 (9.55%), respectively. In comparison with RT-qPCR, additional 14 of RSV positive sample,13 of HRV positive sample and 6 of HMPV positive sample were detected only by mOTNRT-PCR assay, respectively. Sequencing of traditional two-step nested PCR [16] product confirmed these were true positives, suggesting the mOTNRT-PCR assay is more sensitive than RT-qPCR. The originally reported sensitivity of RT-qPCR for detecting RSV [8], HRV [9] in a multiplex PCR format and HMPV in mono RT-qPCR [10] was 5 TCID 50 /ml, not shown and 5–10 copies/reaction, respectively. Even we assume the sensitivities in the original research are comparable to the analytical sensitivities in our study, our data of clinical sensitivities are superior to the those obtained from original reports, as evidenced by the difference in the minimal Ct values for RSV (12.37 vs 17.04), HRV (12.70 vs 19.52) and HMPV (10.79 vs 13.23) between mOTNRT-PCR and corresponding RT-qPCR assays.

mOTNRT-PCR assay can be carried out in one closed tube on real-time PCR machines (CFX96 Touch (Bio-Rad), Roche Light Cycler 480 or ABI 7900HT) with similar results (data not shown), indicating the wide adaptability of mOTNRT-PCR assay. The total detection time is 2.7 h for 96 samples and the cost for one sample is approximately $3.9 (excluding nucleic acid extraction), which is about one thirds of that of monoRT-qPCR assays for detection of RSV, HRV and HMPV in separate individual reaction.

Viral co-infection has been described previously in 5–45% of infected patients in different studies [23–25]. Co-infection of RSV and HRV were the most frequently found which was consistent with the previous report [25]. However, the clinical severity was vague in patients with single or multiple respiratory virus infection in this study due to the limited access to the clinical information. Future work includes further evaluation of mOTNRT-PCR assay using a large number of samples from different population and integrates the clinical data for more comprehensive analysis,which may be beneficial to the patient’s clinical treatment and prognosis.

## Conclusions

We established a mOTNRT-PCR assay allowing simultaneous detection of RSV, HRV, and HMPV. The mOTNRT-PCR assay offers the advantages of higher sensitivity, and more cost-effectiveness than individual RT-qPCR assay, which might be of great potential for clinical use.

## Methods

### Clinical samples

Totally, 398 nasopharyngeal aspirates (NPAs) were collected from inpatients with respiratory tract infections at Children’s Hospital of Hebei, China, between July, 2017 and February, 2018. Of those 167 (41.96%) were female and 231 (58.04%) were male. Ages were ranged from 36 days to 11 years old and 386 (96.98%) were under 5 years old. 1.8 ml of nasopharyngeal aspiratewas collected in 2 ml of transport mediumand stored at − 80 °C. And the study was conducted with the approval of the Ethics Committee of Children’s hospital of Hebei Province, and written informed consents were obtained from the children’s parents.

### Nucleic acid extraction

Total RNA was extracted from 200 μL of clinical sample using the QIAamp Viral RNA Mini Kit (Qiagen, Hilden, Germany). The extracts were eluted into 50 μL RNase-free water and stored at − 80 °C.

### Primers and probes design for mOTNRT-PCR

Inner primer and probe sequences were derived from previously published RT-qPCR assays targeting nucleoprotein gene for RSV [8], the 5′untranslated region for HRV [9] and the nucleoprotein gene for HMPV [10]. The outer primer sequences of RSV, HRV and HMPV were derived from previously published [8–10, 14, 26, 27] with base modifications by LNA [17]. LNA modification can increase maximum annealing temperature of primers [28].

Primers and probes were tested to determine whether they could be used together in a multiplex assay. The specificity of these primer and probe sequences were tested by the BLAST (http://blast.ncbi.nlm.nih.gov/Blast.cgi). And we use oligo7 to evaluate the hairpin of internal primers, primer-dimer potential, G-C content, and melting temperatures of the primers and probes. The primers and probes were obtained from Sangon Biotech (Shanghai, China). The fluorescent reporter dyes for RSV, HRV and HMPV were FAM, Cy5 and VIC, respectively. The sequences of primer and probe are showed in Table 4.

**Table 4:** Primers and probes used in this work

Virus	Oligo	Sequence (5’to 3’)	Primer lengths(bp)	Product size(bp)	Gene	References
RSV	F^a^	CA + CW + GAA + GA + TG + CWAAT+CATAAATTCA	26	374	N	[8, 26]
	R^a^	CW + GA + TC + TRT + CT + CCT + GCTGCTA	21			
	F	CACWGAAGATGCWAATCATAAATTCA	26	89		
	R	GTATYTTTATRGTGTCTTCYCTTCCTAACC	30			
	Probe	FAM-TAATAGGTATGTTATATGCKATGTC-BHQ1	25			
HRV	F^a^	HC + AA+GYA + CTTCT+GTYWCCCCSG	22	397	5′UTR	[9, 27]
	R^a^	GA + AA+CAC + GGA + CA + CCCAAAGTAGT	23			
	F	TGGACAGGGTGTGAAGAGC	19	144		
	R	CAAAGTAGTCGGTCCCATCC	20			
	Probe	Cy5-TCCTCCGGCCCCTGAATG-BHQ3	18			
HMPV	F^a^	CATATAAG+CA + T + G + C + TA + TATTAAAA+GAGTCTC	30	475	N	[10, 14]
	R^a^	GT + GAATATTAA+G + G + CA + C + CTACACATAATAARA	31			
	F	CATATAAGCATGCTATATTAAAAGAGTCTC	30	163		
	R	CCTATTTCTGCAGCATATTTGTAATCAG	28			
	Probe	VIC-TGYAATGATGAGGGTGTCACTGCGGTTG-BHQ1	28			

^a^A ‘+’ symbol in front of nucleotides indicates the LNA modification; FAM, 6-carboxyfluorescein; Cy5, Cyanine5; VIC, 2’-chloro-7’-phenyl-1,4-dichloro-6-carboxyfluorescein; BHQ, Black Hole Quencher

### Preparation of plasmid standards

A 374 bp (nt1277–1650, GenBank accession no. MF737204.1), 397 bp fragment (nt84–480, GenBank accession no. EU096006.1) and 475 bp fragment (nt35–509, GenBank accession no.AY145278.1) corresponding to the nucleoprotein gene of RSV, 5′untranslated region of HRV and nucleoprotein gene of HMPV were respectively cloned by TsingKe Biotech Corp (Beijing, China). The recombinant plasmid was quantified using a Qubit ® dsDNA HS Assay Kits (Life technologies Invitrogen). Plasmid copy number was calculated using the following formula: Plasmid copy number = (copy number / μL) = {[6.02 × 10^23^ × plasmid concentration (ng/μL) × 10^− 9^]} / [Plasmid length× 660]. 10-fold dilutions of mixed recombinant plasmid ranging from 10^8^ to 10^0^ copies /μl were used as standards for the sensitivity analysis of mOTNRT-PCR.

### mOTNRT-PCR amplification

For mOTNRT-PCR assay, the reaction mixture was prepared containing: 5 μL of 5 × PCR buffer, 2μLof One Step RT-PCR Enzyme Mix (Qiagen, Hilden, Germany), 1 mM dNTP mix, 0.1μLof RRI (Takara, Dalian, China), which is the inhibitor of RNase activity, 0.1 μL of each of 0.5 μM RSV, HMPV LNA-outer primer mix, 0.25 μL of 0.5 μM HRV LNA-outer primer mix, 1 μL of each of 5 μM RSV, HMPV inner primer mix, 2.5 μL of 5 μM HRV inner primer mix, 0.5 μL of each of 10 μM RSV, HMPV probe, 1.25μLof 10 μM HRV probe, 5 μL of template nucleic acid and RNAse-free water to reach 25 μL. PCR amplification was performed on the CFX96 Touch (Bio-Rad) and the conditions were: 50 °C for 30 min, a 15 min denaturation step at 95 °C, and 10 cycles of 94 °C for 30s, 64 °C for 40s and 72 °C for 40s, followed by 40 cycles of 94 °C for 30 s, 54 °C for 30s and 72 °C for 30s, with fluorescent readings taken at the the annealing phase of the last 40 cycles. Cycle threshold (Ct) values were calculated using the software at the automatic threshold setting. Positive and negative controls were included in each run. The results were defined as positive if the Ct value was not higher than 35.

### Sensitivity and specificity of the mOTNRT-PCR assay

Sensitivity analysis of mOTNRT-PCR assay was carried out using 10-fold dilutions of mixed plasmid ranging from 10^8^ to 10^0^ copies /μl. Multiplex PCR kit (Qiagen, Hilden, Germany) was used in the sensitivity analysis. In a 25 μL reaction system, 12.5 μL of 2 × QIAGEN Multiplex PCR Master Mix, 0.1 μL of each of 0.5 μM RSV, HMPV LNA-outer primer mix, 0.25 μL of 0.5 μM HRV LNA-outer primer mix, 1 μL of each of 5 μM RSV, HMPV inner primer mix, 2.5 μL of 5 μM inner primer mix, 0.5 μL of each of 10 μM RSV, HMPV probe, 1.25 μL of 10 μM HRV probe, 5 μL of mixed plasmid and 0.3 μL RNAse-free water were added. PCR amplification was performed on the CFX96 Touch (Bio-Rad) and the conditions were: a 15 min denaturation step at 95 °C, and 10 cycles of 94 °C for 30s, 64 °C for 40s and 72 °C for 40s, followed by 40 cycles of 94 °C for 30 s, 54 °C for 30s and 72 °C for 30s, with fluorescent readings taken at the the annealing phase of the last 40 cycles.The specificity was evaluated by using 159 RSV, HRV and HMPV-negative by mOTNRT-PCR out of 398 clinical specimens in this study. These specimens were retrospectively tested by Respiratory Pathogen 13 Detection Kit (13× kit) [15].

### Reproducibility of inter-assay and intra-assay

The intra-assay coefficients of variation (CV) of this assay was tested using three plasmids (10^6^, 10^4^ and 10^2^ copies /μl) of each standard plasmid in three replicates and inter-assay reproducibility was tested in three different days within a week. The variability of the Ct values was assessed.

### Detection of clinical samples

The mOTNRT-PCR assay for the detection of RSV, HRV and HMPV was evaluated using a total of 398 nasopharyngeal aspirates (NPAs) selected from children hospitalized with respiratory infection. For comparison, the previously published RT-qPCR assays [8–10] was also performed in parallel.

### Sequencing

Sequencing of traditional two-step nested PCR [16] product was used to resolve discrepant results between the two assays. Briefly, the product was sequenced in TsingKe Biotech Corp (Beijing, China) using both forward and reverse primers, respectively [16] and the sequencing results were compared with the sequences in GenBank for pathogen identification by using the BLAST algorithm.

## Additional file

Additional file 1: The sequence results of RSV. (DOC 142 kb)

Additional file 2: The sequence results of HRV. (DOC 212 kb)

Additional file 3: The sequence results of HMPV. (DOC 212 kb)

## Abbreviations

ADV: Adenovirus
COV: Coronavirus
Ct: Cycle threshold
CV: Coefficients of variation
Flu A: Influenza virus types A
Flu B: Influenza virus types B
HBOV: Human Bocavirus
HMPV: Human metapneumo virus
HRV: Human rhinovirus
LNA: Locked nucleic acid
mOTNRT-PCR: Multiplex one-tube nested real time RT-PCR
MP: Mycoplasma pneumonia
NPAs: Nasopharyngeal aspirates
PCR: Polymerase chain reaction
PIV: Parainfluenza virus
RSV: Respiratory syncytial virus
RT-qPCR: Traditional real time PCR
SNP: Single nucleotide polymorphism
TSP: Temperature switch PCR
