# Supplementary material for: A multiplex one-tube nested real time RT-PCR assay for simultaneous detection of respiratory syncytial virus, human rhinovirus and human metapneumovirus
Source: Virol J. 2018 Oct 30;15:167. doi: 10.1186/s12985-018-1061-0 (PMC6208169; doi:10.1186/s12985-018-1061-0)
Supplement: Supplementary file 1 — The sequence results of RSV. (DOC 142 kb) [file 12985_2018_1061_MOESM1_ESM.doc]

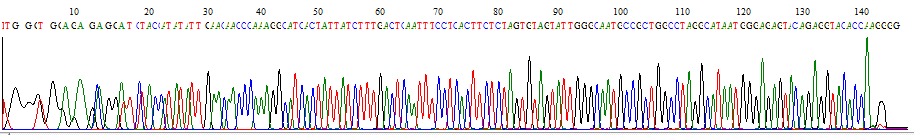


Figure 1 The sequence result of No. 18 RSV positive sample


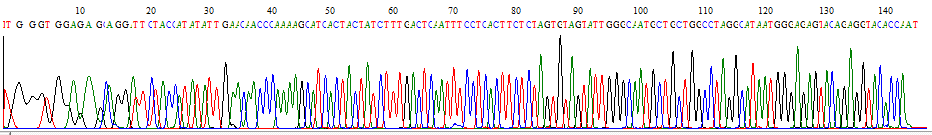


Figure 2 The sequence result of No. 503 RSV positive sample


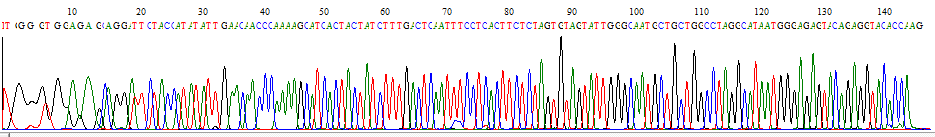


Figure 3 The sequence result of No. 596 RSV positive sample


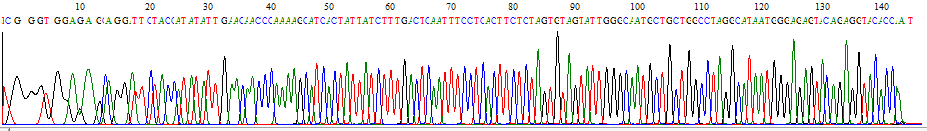


Figure 4 The sequence result of No. 597 RSV positive sample


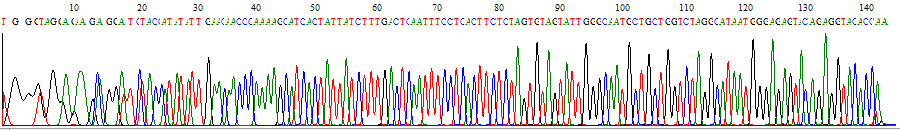


Figure 5 The sequence result of No. 608 RSV positive sample


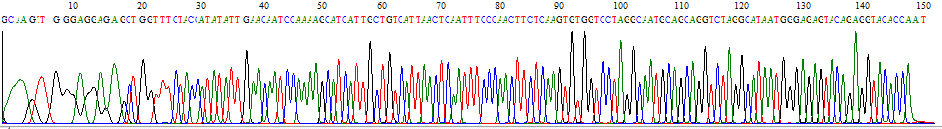


Figure 6 The sequence result of No. 178 RSV positive sample


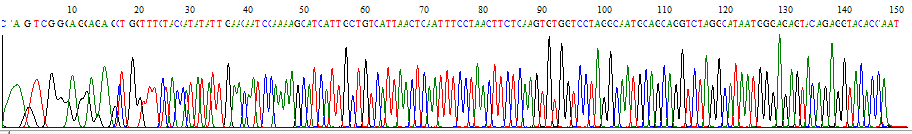


Figure 7 The sequence result of No. 179 RSV positive sample


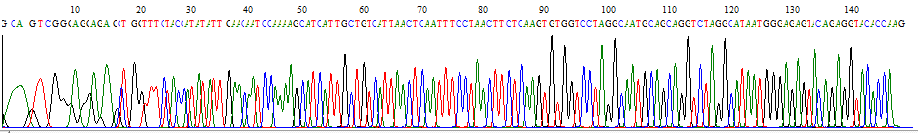


Figure 8 The sequence result of No. 613 RSV positive sample


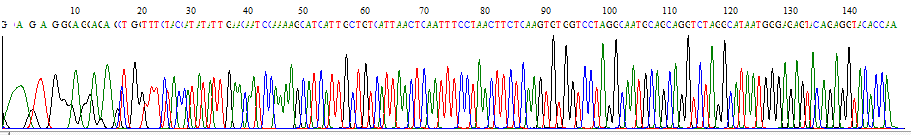


Figure 9 The sequence result of No. 595 RSV positive sample


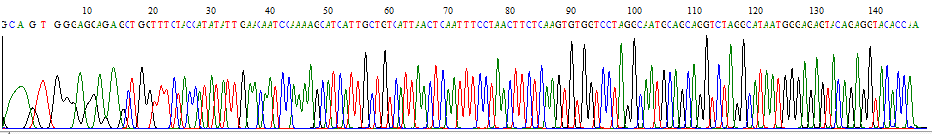


Figure 10 The sequence result of No. 591 RSV positive sample


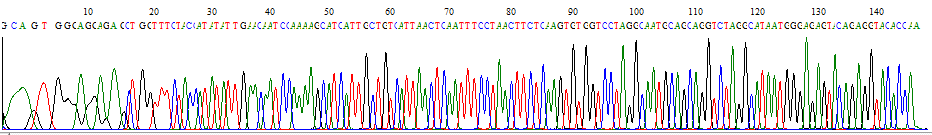


Figure 11 The sequence result of No. 141 RSV positive sample


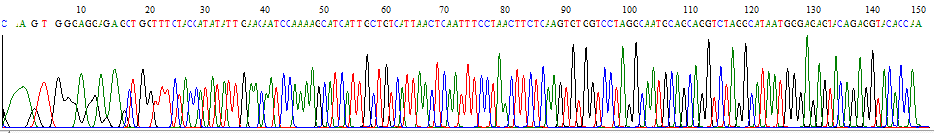


Figure 12 The sequence result of No. 142 RSV positive sample


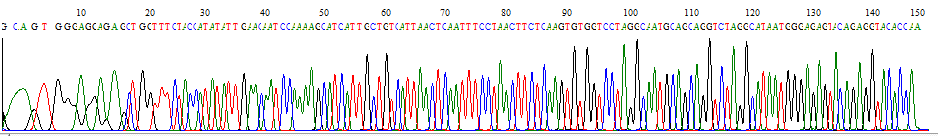


Figure 13 The sequence result of No. 615 RSV positive sample


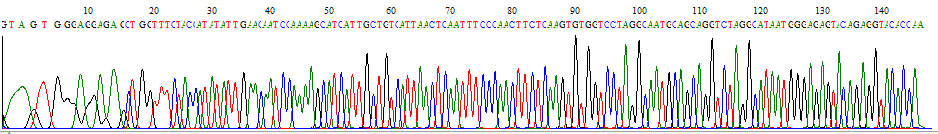


Figure 14 The sequence result of No. 517 RSV positive sample
