# Supplementary material for: A multiplex one-tube nested real time RT-PCR assay for simultaneous detection of respiratory syncytial virus, human rhinovirus and human metapneumovirus
Source: Virol J. 2018 Oct 30;15:167. doi: 10.1186/s12985-018-1061-0 (PMC6208169; doi:10.1186/s12985-018-1061-0)
Supplement: Supplementary file 2 — The sequence results of HRV. (DOC 212 kb) [file 12985_2018_1061_MOESM2_ESM.doc]

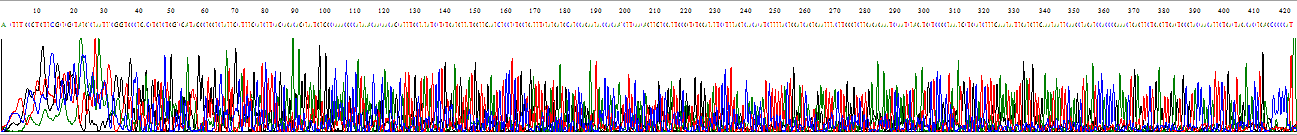


Figure 1 The sequence result of No. 297 HRV positive sample


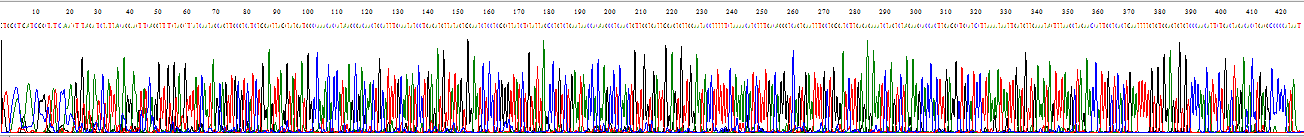


Figure 2 The sequence result of No. 298 HRV positive sample


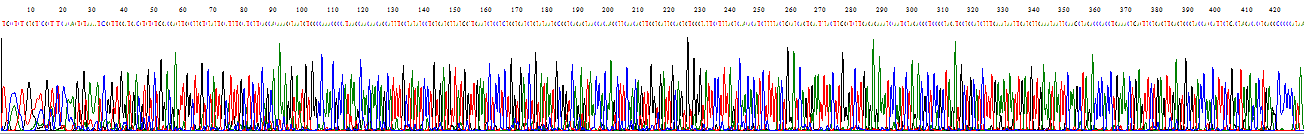


Figure 3 The sequence result of No. 299 HRV positive sample


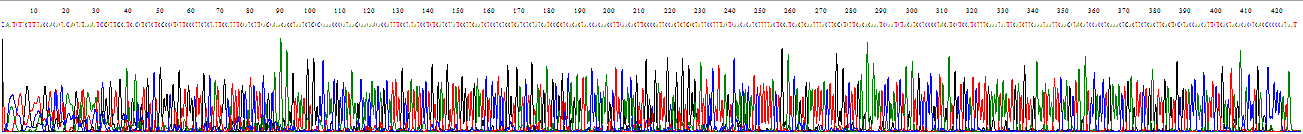


Figure 4 The sequence result of No. 326 HRV positive sample


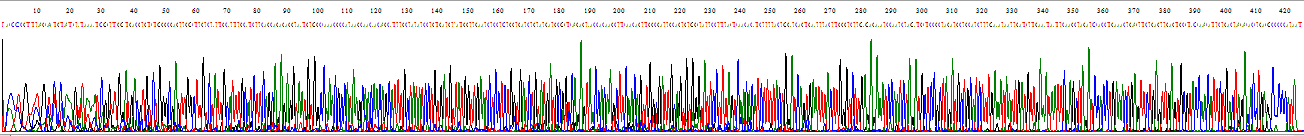


Figure 5 The sequence result of No. 395 HRV positive sample


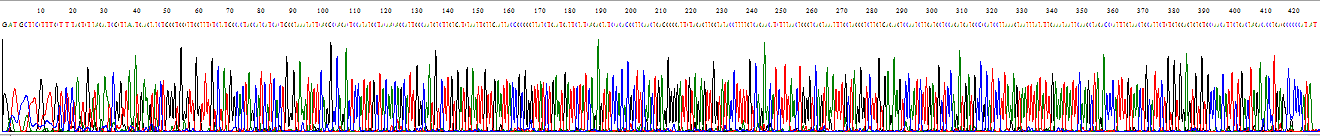


Figure 6 The sequence result of No. 397 HRV positive sample


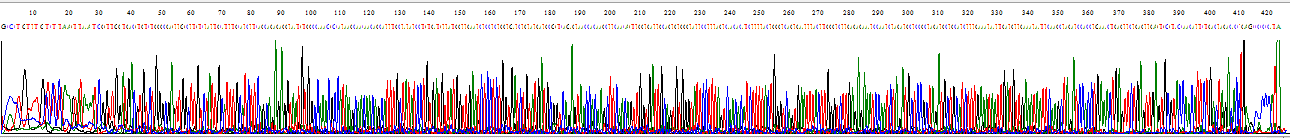


Figure 7 The sequence result of No. 408 HRV positive sample


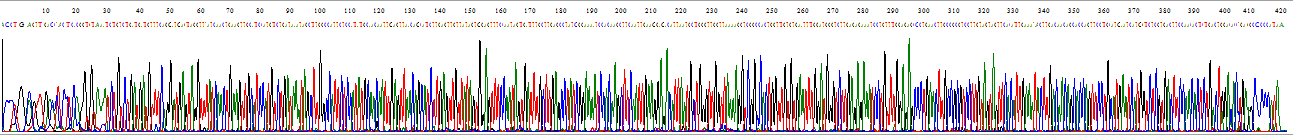


Figure 8 The sequence result of No. 318 HRV positive sample


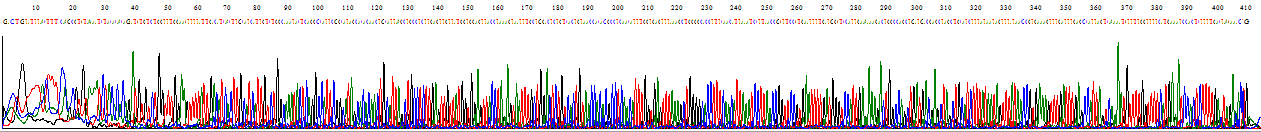


Figure 9 The sequence result of No. 404 HRV positive sample


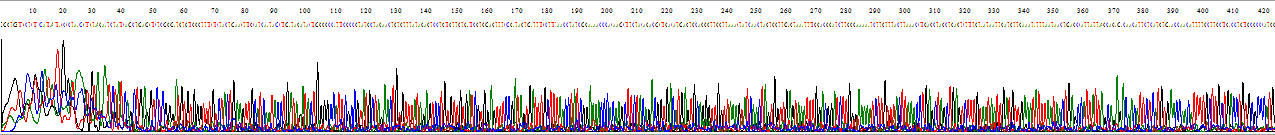


Figure 10 The sequence result of No. 342 HRV positive sample


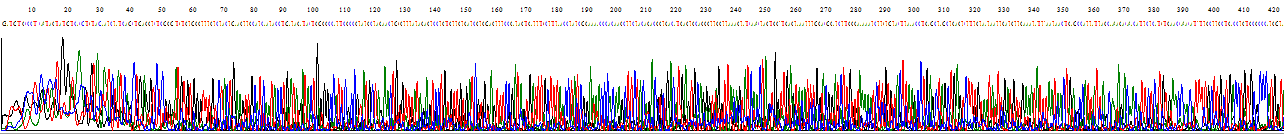


Figure 11 The sequence result of No. 356 HRV positive sample


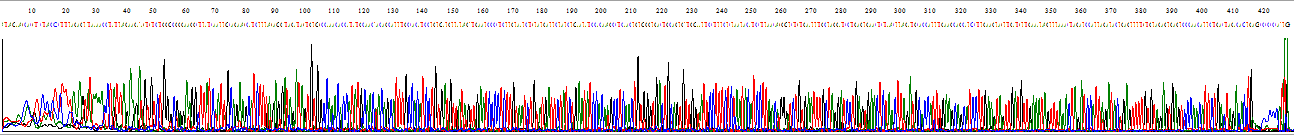


Figure 12 The sequence result of No. 311 HRV positive sample


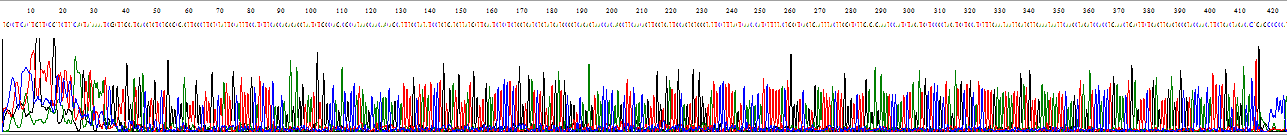


Figure 13 The sequence result of No. 312 HRV positive sample
