# Supplementary material for: A multiplex one-tube nested real time RT-PCR assay for simultaneous detection of respiratory syncytial virus, human rhinovirus and human metapneumovirus
Source: Virol J. 2018 Oct 30;15:167. doi: 10.1186/s12985-018-1061-0 (PMC6208169; doi:10.1186/s12985-018-1061-0)
Supplement: Supplementary file 3 — The sequence results of HMPV. (DOC 212 kb) [file 12985_2018_1061_MOESM3_ESM.doc]

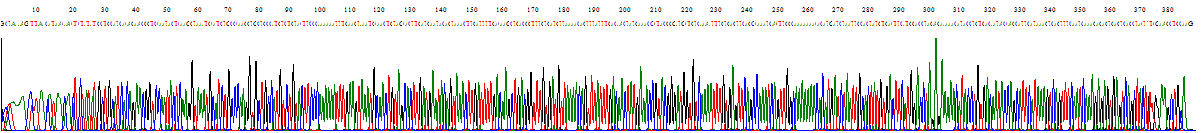


Figure 1 The sequence result of No. 103 HMPV positive sample


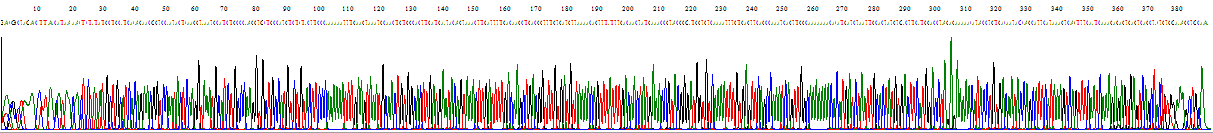


Figure 2 The sequence result of No. 120 HMPV positive sample


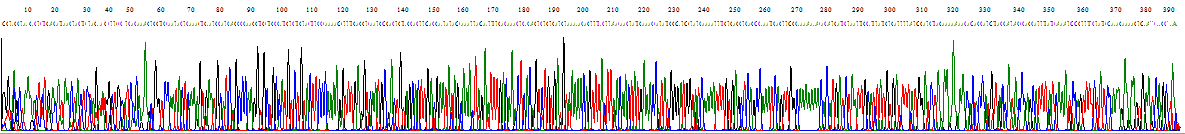


Figure 3 The sequence result of No. 177 HMPV positive sample


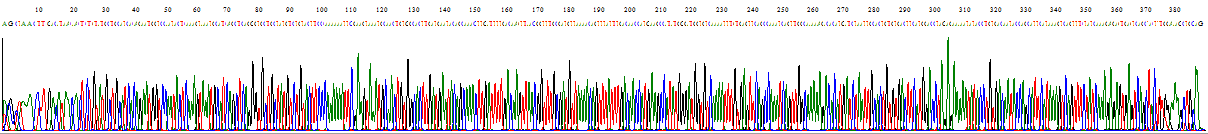


Figure 4 The sequence result of No. 245 HMPV positive sample


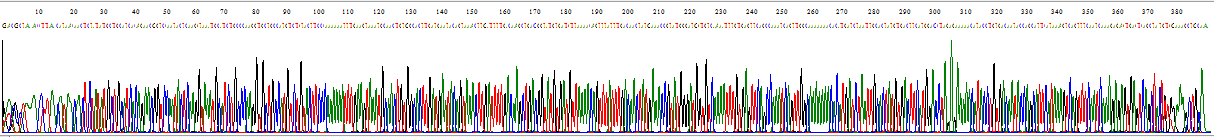


Figure 5 The sequence result of No. 474 HMPV positive sample


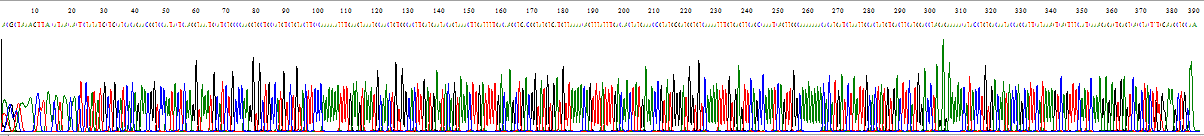


Figure 6 The sequence result of No. 574 HMPV positive sample
